# Case Report: Resection of Giant Endotracheal Hamartoma by Electrosurgical Snaring via Fiberoptic Bronchoscopy in a 9-Year-Old Boy

**DOI:** 10.3389/fped.2021.528966

**Published:** 2021-04-27

**Authors:** Lei Wu, Wei Chen, PengCheng Li, Shuxian Li, Zhimin Chen

**Affiliations:** ^1^Department of Pulmonology, The Children's Hospital, Zhejiang University School of Medicine, National Clinical Research Center for Child Health, Hangzhou, China; ^2^Department of Endoscopy Center, The Children's Hospital, Zhejiang University School of Medicine, National Clinical Research Center for Child Health, Hangzhou, China; ^3^Department of Pediatrics, The First Affiliated Hospital of USTC, Division of Life Sciences and Medicine, University of Science and Technology of China, Hefei, China; ^4^Department of Pediatrics, Affiliated Hospital of Nantong University, Nantong, China

**Keywords:** endotracheal lesions, hamartoma, therapeutic bronchoscopy, airway tumors, case report

## Abstract

Endotracheal hamartomas are rarely encountered in children. The symptoms of endotracheal hamartoma may include cough, dyspnea, hemoptysis, chest pain, purulent sputum, and fever. The non-specific symptoms often result in a delayed diagnosis. Among the various treatments of this rare disease, surgical resection seems to be the most widely used, while endoscopic treatment is rarely described. Herein, we describe the case of a 9-year-old boy with an endotracheal hamartoma that was successfully excised by electrosurgical snaring via fiberoptic bronchoscopy (FB). The resection of select benign endotracheal tumors in children can be conducted using electrocautery, which can be regarded as an alternative therapy to bronchotomy.

## Introduction

Benign lung tumors represent <1% of all lung tumors; among these, hamartomas have an incidence of 0.025–0.32% in the adult population (1). Although still an uncommon occurrence, hamartoma is the most common type of benign pulmonary tumor, accounting for an estimated 77% of benign lung nodules and 8% of solitary lung lesions (2, 3). Most hamartomas occur in the peripheral parenchyma, but have in rare cases been present in the trachea or bronchi. A previous study reported that the hamartoma was in an endobronchial location in only 1.4% of 215 patients with hamartomas (4). Although hamartomas may be found at any age, the age at presentation is mostly between 40 and 60 years; primary pulmonary, tracheal, or bronchial hamartomas are significantly rarer in the pediatric population (2, 5). The most frequent clinical symptoms of tracheal or endobronchial hamartomas are fever, cough, hemoptysis, purulent sputum, dyspnea, and pain due to tracheal or bronchial obstruction. Tracheal or endobronchial hamartomas are traditionally treated by thoracotomy with bronchotomy or lung resection. At present, the first-line widely recommended approach in adults is endoscopic treatment; however, similar treatments have rarely been reported in children (6). We herein describe a case in which electrosurgical snaring via fiberoptic bronchoscopy (FB) was applied to successfully resect a rare tracheal hamartoma in a young boy, avoiding the need for bronchotomy.

## Case Presentation

A 9-year-old boy was referred to our hospital with a 20-day history of cough, stridor, and shortness of breath. He had showed a poor clinical response to conventional therapy with antibiotics and systemic steroids at a local hospital. He was previously healthy with no relevant family history. A computed tomography (CT) scan performed at a local hospital revealed a homogeneous mass obstructing the trachea. During preparation for rigid bronchoscopy (RB) at the local hospital, he was found to be intolerant to general anesthesia and his dyspnea exacerbated. He was transferred to our hospital immediately. On admission to our hospital, the patient appeared dyspneic and had a characteristic biphasic stridor, with a percutaneous oxygen saturation of 93% under mask oxygen inhalation, body temperature of 37.7°C, pulse rate of 162 beats/min, respiration rate of 34 breaths/min, and blood pressure of 127/74 mmHg.

**Figure 1:**
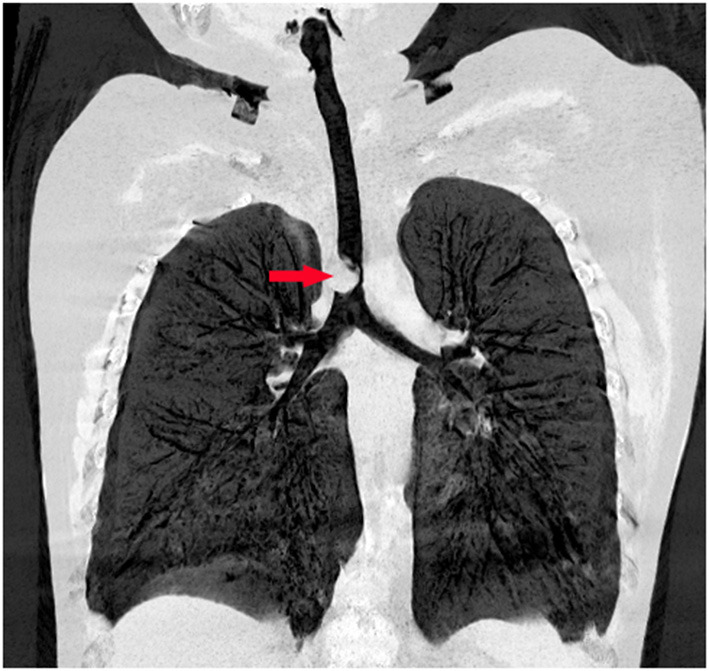
Preoperative chest computed tomography reveals an endotracheal tumor nearly totally obstructing the tracheal lumen.

On day 2, contrast-enhanced multidetector CT revealed a mass lesion with a fat density that was nearly totally obstructing the tracheal lumen (Figure 1). The multidisciplinary consensus was to proceed with bronchoscopy to obtain a definitive diagnosis to guide management and determine the prognosis. After being fully informed about the surgical options and possible complications related to different surgical modalities, the patient's parents elected to have the mass removed by an electrocautery snare with FB. A rigid bronchoscope (Karl Storz, Germany), argon plasma coagulation (APC) (ERBE VIO200D+APC2), cryotherapy (ERBOKRYO CA), and Holmium: YAG laser (Dahua DHL-1, China) were available if necessary. The parents also agreed that if the treatment failed, surgical resection would be carried out immediately. Written informed consent for the interventional procedures was obtained from the patient's parents.

**Figure 2:**
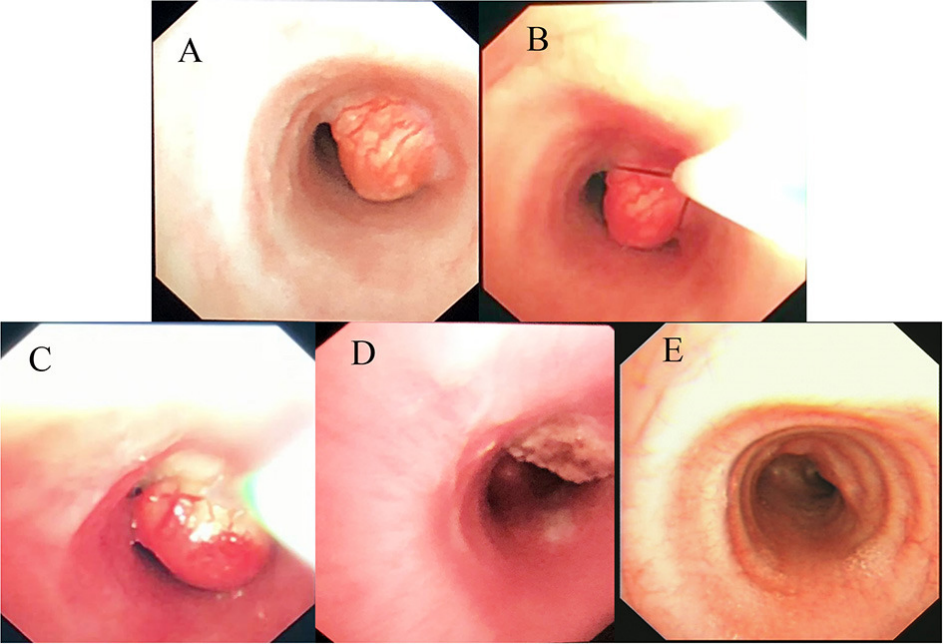
**(A)** Fiberoptic bronchoscopy image of the large mass covered by smooth mucosa in the mid-tracheal region. **(B)** Placing the loop of the snare around the base of the mass. **(C)** The process of mass removal by electrocautery snare. **(D)** Most of the mass is removed by electrosurgical snaring under bronchoscopy. **(E)** Photograph showing a slight protrusion at the position of the incision at 1 year post-operatively.

**Figure 3:**
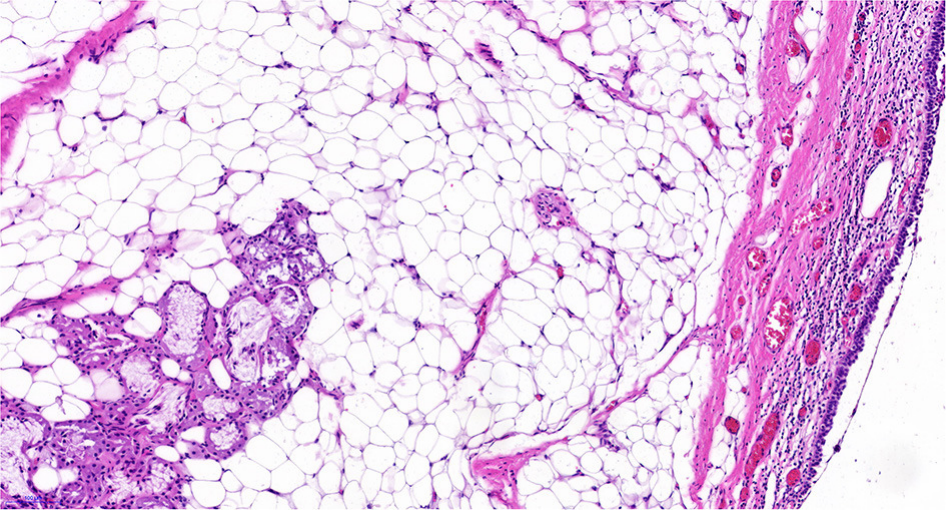
The tumor was a hamartoma largely comprising mature fat cells.

**Figure 4:**
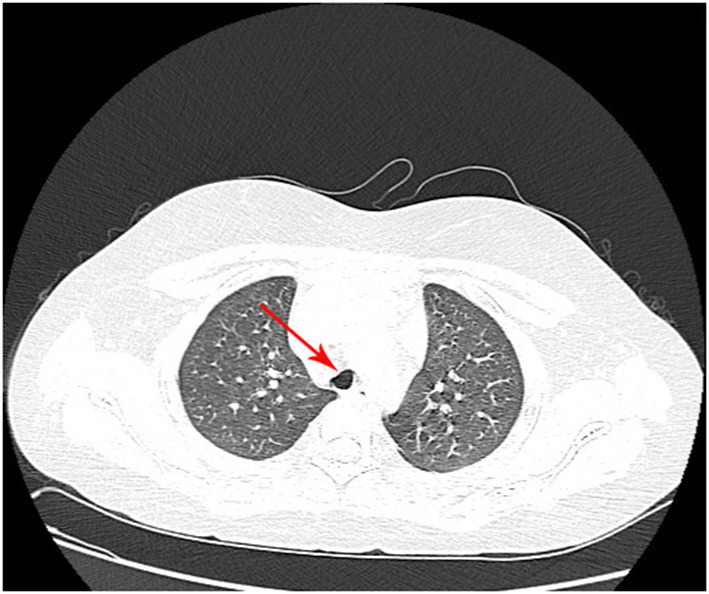
Chest computed tomography at 1 year postoperatively shows that the anterior wall of the trachea has collapsed slightly.

The mass resection was performed on day 3. Pulmonologists, otolaryngologists, thoracic surgeons, anesthesiologists, and pediatric intensive care unit doctors were all in the operating room to ensure the success of the treatment and the safety of the patient. General anesthesia was induced with 8% sevoflurane. After the intravenous administration of 2 mg of midazolam, a laryngeal mask airway (LMA) was inserted to maintain an open airway. Intraoperatively, anesthesia was maintained with 3% sevoflurane. Based on the depth of anesthesia, 10 mg of pentazocine and 50 mg of propofol were intravenously administered intraoperatively. A swivel adapter was used to connect the proximal end of the laryngeal mask to the T-piece anesthesia system. A flexible fiberoptic bronchoscope (Olympus 260; external diameter: 4.9 mm, working channel: 2 mm) operated by an experienced pulmonologist was inserted via the swivel adapter. A large mass covered by smooth mucosa with a stalk was obstructing approximately 85% of the trachea in the mid-tracheal region (Figure 2A). A flexible 1.9-mm electrocautery snare (Olympus CD-6C-1) was then introduced through the working channel of the bronchoscope. After placing the loop of the snare around the base of the mass (Figure 2B), the electrocautery snare was used in blend mode at 30 W to cut and coagulate the base of the mass (Figure 2C). The mass was resected without evidence of bleeding or perforation (Figure 2D). The inspired oxygen concentration was maintained at 25% to avoid potential combustion during the interventional procedure. Due to the broad base of the mass, part of the remaining tissue at the root was removed with biopsy forceps to reduce the amount of residual tissue. Postoperatively, the patient recovered completely and was discharged home on day 8. The pathologic examination suggested that the mass was a tracheal hamartoma predominantly constituted of mature fat cells (Figure 3). The patient's condition remained stable for 1 year following the interventional therapy. The patient returned to the hospital for repeat chest CT examination and bronchoscopy at 1 year postoperatively (Figure 4). There was a slight protrusion at the position of the incision (Figure 2E).

## Discussion

Primary endobronchial and endotracheal tumors are uncommon in childhood. Of these, benign tumors consist of inflammatory pseudotumors (plasma cell granulomas), papillomas, hamartomas, mucus gland tumors, leiomyomas, and hemangiomas (7). Malignant tumors include mucoepidermoid carcinomas, carcinoids, adenoid cystic carcinomas, and bronchial adenomas (an obvious misnomer). The most common benign primary pulmonary tumors in children are inflammatory pseudotumors, followed by hamartomas (7).

Hamartomas in the lung are slow and abnormal growing neoplasms that are defined as a tumor-like malformation in which there is abnormal mixing of the normal components of the organ. In other words, a pulmonary hamartoma is a rare benign tumor that originates from the bronchial primitive mesenchymal tissue and can differentiate into various mature mesenchymal components (8). The histogenesis of hamartoma remains unclear. However, there are four etiologic theories: neoplasia, hyperplasia of a normal structure, congenital malformation, and response to inflammation. Both intraparenchymal and endobronchial hamartomas generally contain cartilage, bone, fat, and muscle tissue. Endobronchial hamartomas tend to have more fat than intraparenchymal hamartomas, which may be attributed to the relative abundance of fat in the tracheal walls as opposed to the lung parenchyma (9). This may partially explain why the mass in our patient was composed mainly of fatty tissue.

Patients with endotracheal hamartomas are generally asymptomatic, at least in the preocclusive early phase. The presenting symptoms including cough, dyspnea, hemoptysis, chest pain, purulent sputum, and fever are induced by tracheobronchial obstruction and can lead to recurrent pneumonia (10). As the clinical symptoms are not specific and depend on the size and localization of the tumor, endotracheal hamartomas are often misdiagnosed as more benign conditions, delaying definitive treatment (6, 11). Clinically, the differential diagnoses include aspirated foreign bodies, ectopic thyroid tissue, mucosal webs, cysts, as well as malignancies, including lipoma and lymphoma. With the popularization of CT and magnetic resonance imaging (MRI), it is often possible to determine a diagnosis or narrow the differential diagnosis list. Computed tomography (CT) is sensitive in diagnosing fat collections or calcifications. Fortunately, the hamartoma in our case mainly contained fat. In addition, the present patient was in a sitting breathing state because of airway obstruction caused by the hamartoma before surgery and could not tolerate lying flat for a long time for MRI examination. For these two reasons, we did not perform MRI. However, some hamartomas containing neither fat nor calcification are difficult to differentiate from malignant tumors. Magnetic resonance imaging is an excellent diagnostic tool for the evaluation of soft tissue tumors and has great diagnostic value for detecting the typical cleft-like structure in a pulmonary lesion when neither fat nor calcification is present on CT (12). Therefore, MRI may serve as an adjuvant or alternative diagnostic method to CT for thoracic lesions. However, the definitive diagnosis of hamartoma depends on the histological examination.

Surgical removal of the tumor in older patients should be done only when the tumor is symptomatic or if malignant transformation is suspected. In contrast to adults, peripheral and central hamartomas in children can cause marked symptoms and require prompt surgical removal. The traditional treatment is pneumonectomy or thoracotomy combined with bronchial resection in children (13). However, the first-line widely recommended therapy in adults is endoscopic treatment due to the benign nature of this disease with extremely rare malignant transformation and low recurrence rate (14). Our pediatric patient underwent successful airway recanalization via a flexible bronchoscope. However, due to the low incidence of this condition, experience with the endotracheal resection of hamartomas is relatively limited and there is no standard management for endotracheal hamartoma. Interventional bronchoscopic techniques utilized to manage airway lesions include electrosurgical snaring, APC, cryotherapy, and laser ablation. In the present case, the use of snare electrocautery minimized the bleeding risk by simultaneously cauterizing the base of the tumor during resection. Additionally, considering the unknown nature of the lesion at the time of presentation, we did not perform cryotherapy and APC to achieve hemostasis and destroy the residual tissue immediately after electrocautery. The bronchoscopic treatment options are dependent on the location and characteristics of the airway lesion, physician's preference, expertise, and device availability (6).

Rigid bronchoscopy is probably the best conduit for managing endotracheal lesions, although bronchoscopy via a LMA is a viable option. However, it is important to note that the parents of pediatric patients tend to choose FB. Because the patient could not tolerate anesthesia at the local hospital, he was unable to undergo RB there. Therefore, we chose to use bronchoscopy via a LMA rather than RB. The advantage of LMA general anesthesia is that it enables control of the patient's breathing and provides a large space for endoscopic operation. If it had been difficult to remove the tumor under FB, we would have attempted mechanical debulking under RB. If the patient had not been able to tolerate laryngeal mask anesthesia, we would have attempted to resect the mass via FB under tracheal intubation anesthesia. If we had been unable to remove the mass via FB under tracheal intubation anesthesia, the thoracic surgeon would have performed an open thoracotomy. The extracorporeal membrane oxygenation team was ready to provide emergency treatment if the vital signs became unstable. The management of pulmonary hamartoma might be highly center- and patient-dependent. There is no gold standard due to the rare nature of the lesion and lack of uniformity in expertise among centers in managing such cases. Therefore, a large prospective clinical trial is needed.

The electrocautery snare has the ability to sever a large mass quickly with concomitant hemostasis. The advantages of electrosurgery via FB are the provision of larger tissue specimens for more accurate histological diagnosis, recanalization of the involved airway, better assessment of the tumor base, more accurate surgical planning, stabilization of the patient to enable them to undergo surgery in a better general condition, and minimization of the bleeding risk by simultaneous cauterization of the base of the tumor during resection. Despite the partial residual tissue (Figure 2D), our patient's symptoms were relieved immediately after electrocautery, indicating that electrosurgical snaring is a potentially useful method with which to manage endotracheal hamartoma. Once the emergency situation was handled, the next step was to evaluate the need for further management depending on the histopathology. Although we advised further bronchoscopic debulking (e.g., cryotherapy and APC) of the residual mass (Figure 2D) based on the histopathologic result, the parents chose serial clinic visits, imaging, and airway inspections rather than bronchoscopic debulking. The patient remained recurrence- and symptom-free and the airway remained patent following bronchoscopic surveillance at the 1-year follow-up (Figures 2E, 4); this indicates that the outcome of electrosurgical snaring via FB for tracheal hamartoma appears to be satisfactory, although the long-term survival remains to be assessed. Hence, our experience suggests that bronchoscopic treatment can be considered as a bridging therapy to definitive treatment.

As hamartoma is a benign neoplasm, endoscopic treatment is now widely recommended as the first-line approach. Theoretically, hamartoma can be managed endoscopically in all age groups. However, the diameter of the electrosurgical snare and the working channel of the flexible bronchoscope limit the wide application of this technique. The diameter of the flexible electrocautery snare is 1.9 mm, which can pass through the working channel of the flexible bronchoscope BF-P260 (external diameter: 4.0 mm, working channel: 2.0 mm). As the smallest flexible bronchoscope with a 2.0-mm working channel, our experience suggests that the Olympus P260 can be used in children older than 2 years. Thus, electrosurgical snaring might be useful for endoscopically managing tumors in children ≥2 years.

The common complications of electrosurgical snaring include bleeding, perforation, and endotracheal burns (15). The main reason for bleeding during electrocautery snaring is that the snare shrinks too fast when cutting the tumor, which makes the electrical cut a simple mechanical cut, leading to tumor tearing and bleeding. The optimal strategy to prevent bleeding is to cut the mass slowly, so that there is enough time to cauterize the basal tissue while cutting the mass, and the basal tissue is coagulated and denatured. The main reason for airway wall perforation is that the direction of electric cutting deviates from the direction of the bronchus during the treatment process. Therefore, a good visual field should be maintained to avoid the cutting angle deviating from the direction of the bronchus. Using APC cauterization or cryotherapy at the mass root can reduce the risk of tumor recurrence. In the present case, we did not perform mass root interventional therapy because the airway obstruction was relieved after mass resection via the electrocautery snare, and the pathological diagnosis was not clear at that time. Although a small part of the tumor remained after resection, the patient had no symptoms, and the parents were not willing to consent to further thoracotomy. If the tumor continues to grow, thoracotomy is the best option.

Although interventional bronchoscopy is a mature technique in adults, it is seldom used in children. However, our case suggests that under careful preparation, interventional bronchoscopy can be safe and beneficial in pediatric patients.

## Conclusion

Endotracheal hamartoma is a rare benign tumor, and bronchoscopic therapy is recommended as the first-line treatment, although surgical resection may be necessary under certain conditions. Clinicians should consider the presence of tracheal lesions such as hamartomas in pediatric patients who obtain no significant improvement of respiratory symptoms after conventional treatment.

## Data Availability Statement

The original contributions presented in the study are included in the article/supplementary material, further inquiries can be directed to the corresponding author/s.

## Ethics Statement

Ethical review and approval was not required for the study on human participants in accordance with the local legislation and institutional requirements. The patients/participants (legal guardian/next of kin) provided written informed consent to participate in this study.

## Author Contributions

ZC supervised research work. LW and SL participated in collecting information of this case and searched for literatures. All authors participated in the interpretation of the data, contributed to the article, and approved the submitted version.

## Conflict of Interest

The authors declare that the research was conducted in the absence of any commercial or financial relationships that could be construed as a potential conflict of interest.

## References

[1] Murray J Kielkowski D Leiman G. The prevalence and age distribution of peripheral pulmonary hamartomas in adult males. An autopsy-based study. S Afr Med J. (1991) 79:247–9. 2011801

[2] Ahmed S Arshad A Mador MJ. Endobronchial hamartoma; a rare structural cause of chronic cough. Respir Med Case Rep. (2017) 22:224–7. 10.1016/j.rmcr.2017.08.019 28913162 PMC5587872

[3] Lu Z Qian F Chen S Yu G. Pulmonary hamartoma resembling multiple metastases: a case report. Oncol Lett. (2014) 7:1885–8. 10.3892/ol.2014.2043 24932253 PMC4049732

[4] Gjevre JA Myers JL Prakash UB. Pulmonary hamartomas. Mayo Clin Proc. (1996) 71:14–20. 10.4065/71.1.14 8538225

[5] Saadi MM Barakeh DH Husain S Hajjar WM. Large multicystic pulmonary chondroid hamartoma in a child presenting as pneumothorax. Saudi Med J. (2015) 36:487–9. 10.15537/smj.2015.4.10210 25828288 PMC4404485

[6] Pio L Varela P Eliott MJ Couloigner V Guillen Burrieza G Paraboschi I. Pediatric airway tumors: a report from the International Network of Pediatric Airway Teams (INPAT). Laryngoscope. (2019) 130:E243–51. 10.1002/lary.28062 31090942

[7] Jain V Goel P Kumar D Seith A Sarkar C Kabra SK. Endobronchial chondroid hamartoma in an infant. J Pediatr Surg. (2009) 44:e21–3. 10.1016/j.jpedsurg.2009.06.011 19735804

[8] Tomashefski JF. Benign endobronchial mesenchymal tumors: their relationship to parenchymal pulmonary hamartomas. Am J Surg Pathol. (1982) 6:531. 10.1097/00000478-198209000-00005 7149093

[9] Gaerte SC Meyer CA Winer-Muram HT Tarver RD Conces DJ Jr. Fat-containing lesions of the chest. Radiographics. (2002) 22:S61. 10.1148/radiographics.22.suppl_1.g02oc08s61 12376601

[10] Mondello B. Giant endobronchial hamartoma resected by fiberoptic bronchoscopy electrosurgical snaring. J Cardiothorac Surg. (2011) 6:97. 10.1186/1749-8090-6-97 21838930 PMC3170318

[11] Varela P Pio L Torre M. Primary tracheobronchial tumors in children. Semin Pediatr Surg. (2016) 25:150–5. 10.1053/j.sempedsurg.2016.02.013 27301601

[12] Park KY Kim SJ Noh TW Cho SH Lee DY Paik HC. Diagnostic efficacy and characteristic feature of MRI in pulmonary hamartoma: comparison with CT, specimen MRI, and pathology. J Comput Assist Tomogr. (2008) 32:919–25. 10.1097/RCT.0b013e31815abed4 19204455

[13] Borro JM Moya J Botella JA Padilla JD Canto A Paris F. Endobronchial hamartoma. Report of 7 cases. Scand J Thorac Cardiovasc Surg. (1989) 23:285–7. 10.3109/14017438909106011 2617250

[14] Sedat A Levent D Levent KC Erdogan L Sinem T Nur S. Resection of giant endobronchial hamartoma by electrocautery and cryotherapy via flexible bronchoscopy. Tüberk Toraks. (2007) 55:390–4. 18224508

[15] van Boxem TJ Westerga J Venmans BJ Postmus PE Sutedja TG. Tissue effects of bronchoscopic electrocautery: bronchoscopic appearance and histologic changes of bronchial wall after electrocautery. Chest. (2000) 117:887–91. 10.1378/chest.117.3.887 10713021

